# Evaluation of Preoperative Left Ventricular Relative Wall Thickness for Predicting Postoperative Acute Kidney Injury in Elderly Hip Fracture Patients

**DOI:** 10.3390/jcm15031115

**Published:** 2026-01-30

**Authors:** İmran Ceren, Dilek Kalaycı, Arif Timuroğlu, Kemal Göçer, Yusuf Ziya Şener, Eser Açıkgöz, Fadime Bozduman Habip, Coşkun Ulucaköy

**Affiliations:** 1 Department of Cardiology, Dr Abdurrahman Yurtaslan Ankara Oncology Training and Research Hospital, 06200 Ankara, Türkiye; 2 Department of Anaesthesia and Reanimation, Dr Abdurrahman Yurtaslan Ankara Oncology Training and Research Hospital, 06200 Ankara, Türkiyeariftimuroglu@yahoo.com (A.T.); 3 Department of Cardiology, HG Hospital, 46050 Kahramanmaraş, Türkiye; 4 Thoraxcenter, Erasmus MC, 3015 GD Rotterdam, The Netherlands; 5 Department of Orthopedics and Traumatology, Dr Abdurrahman Yurtaslan Ankara Oncology Training and Research Hospital, 06200 Ankara, Türkiye

**Keywords:** left ventricular remodeling, elderly patients, hip fracture surgery, acute kidney injury

## Abstract

**Objectives:** This study aimed to explore the association between left ventricular relative wall thickness (RWT) and postoperative acute kidney injury (AKI) in elderly patients who underwent hip fracture surgery. Additionally, we evaluated the prognostic value of RWT for postoperative clinical outcomes in this high-risk group. **Methods:** This prospective study included 131 patients aged ≥ 65 years who underwent surgery for femoral neck or intertrochanteric hip fractures. Preoperative echocardiographic parameters, including RWT, were recorded and their associations with postoperative AKI were analyzed. Postoperative cardiovascular complications and clinical outcomes were assessed. **Results:** Postoperative AKI occurred in 19.1% of patients and was significantly associated with higher in-hospital mortality (20% vs. 5.6%; *p* = 0.036). Patients who developed AKI had significantly higher RWT (0.54, 0.503, *p* = 0.048, respectively). Receiver operating characteristic (ROC) curve analysis was performed for preoperative echocardiographic parameters, such as interventricular septum (IVS), posterior wall thickness (PWT), left atrium (LA) diameter, and RWT, to evaluate their predictive ability for AKI. The area under the curve (AUC) values were 0.645 for IVS, 0.632 for PWT, 0.713 for LA diameter, and 0.628 for RWT (all *p* < 0.05). Although LA diameter had the highest AUC, RWT showed the highest sensitivity (96%) at the cut-off value of 0.435. Subgroup analyses comparing patients with RWT < 0.435 and ≥0.435 showed no significant differences in AKI, mortality, delirium, intensive care unit admission rates, cardiac complications, or ischemic events (all *p* > 0.05). **Conclusions:** Preoperative RWT demonstrated a modest but statistically significant association with postoperative AKI in elderly hip fracture patients with preserved left ventricular ejection fraction. Although its standalone predictive value is limited, RWT may contribute to perioperative risk stratification when interpreted alongside other echocardiographic, clinical, and biochemical parameters in this vulnerable high-risk patient population.

## 1. Introduction

Hip fractures encompass femoral neck and intertrochanteric fractures and are considered serious injuries due to their high mortality rates [1]. The absolute number of such fractures is increasing globally, especially in aging populations [1,2], and is even projected to reach approximately 6 million by 2050 [3]. Despite advancements in surgical management, postoperative outcomes remain a concern. A 30-day mortality rate of 4.3% has been documented after hip fracture surgery, and the one-year mortality rate reaches 18.8% [4]. Identifying high-risk patients before surgery is clinically important, as hip fractures in elderly individuals are associated with high mortality and prolonged functional decline [2,5].

Among the postoperative complications of hip fractures, serious conditions such as venous thrombosis, pulmonary embolic events, acute kidney injury (AKI), and myocardial infarction are notable [6,7]. AKI is the sudden decrease in glomerular filtration rate (GFR) and significantly contributes to morbidity and mortality in the postoperative period after hip fracture [7,8]. Its prevention is crucial, as it can predispose to chronic kidney disease (CKD) and increase mortality risk [8]. The incidence of AKI following elderly hip fracture surgery varies in the literature, ranging between 9.8% and 20.6% [9]. This complication prolongs hospital stays and negatively affects postoperative clinical outcomes [9,10]. Recovery from AKI is often poor in individuals over the age of 65, and the condition frequently progresses to chronic kidney failure in this population [10]. The evidence reinforces the importance of closely monitoring for postoperative AKI in elderly hip fracture patients and managing modifiable risk factors effectively.

Although left ventricular ejection fraction (LVEF) is widely used to assess systolic function, its association with postoperative morbidity is mainly evident when LVEF is at specific thresholds, such as <40% or <30% [11,12]. However, most patients undergo high-risk surgeries with a normal LVEF, and it has been shown that LVEF may inadequately reflect cardiac function, particularly in elderly and hypertensive individuals [13,14]. In this context, left ventricular relative wall thickness (RWT) has emerged as an alternative echocardiografic parameter that can reveal structural cardiac abnormalities independently of LVEF [14,15,16]. An increase in RWT reflects cardiac remodeling due to chronic pressure overload [17]. A value of 0.42 is considered the threshold for RWT; this value generally defines the limit of left ventricular concentric hypertrophy and 0.32 for eccentric hypertrophy [17]. Even in patients with normal LVEF, elevated RWT has been associated with reduced myocardial contractility, increased risk of heart failure, and higher long-term mortality [15,18].

Previous research has demonstrated an association between elevated RWT and an increased risk of postoperative AKI in patients undergoing high-risk non-cardiac surgeries [19]. Similar findings have been reported in oncologic thoracic surgery populations [20]. Although several studies have reported an association between RWT and postoperative AKI across different surgical settings, data specifically focusing on elderly patients undergoing hip fracture surgery remain limited. In this regard, this study aimed to evaluate the association between preoperative echocardiographic parameters and the development of postoperative AKI in elderly patients undergoing surgery for hip fracture. To the best of our knowledge, this is the first prospective study to evaluate the association between preoperative RWT and postoperative AKI in this vulnerable and high-risk patient group. Furthermore, it is innovative and distinct in that it only included elderly patients over the age of 65. Through this study, we hypothesize that increased RWT may predict postoperative AKI in elderly hip fracture patients with preserved LVEF. This easily implemented preoperative method may enable the identification of patients at risk for postoperative AKI and improve patient outcomes.

## 2. Patients and Methods

This study was designed prospectively and included patients over the age of 65 who underwent surgery for hip fractures at our orthopedic clinic between November 2023 and April 2025. The definition of hip fracture referred to intertrochanteric and femoral neck fractures.

**Figure 1:**
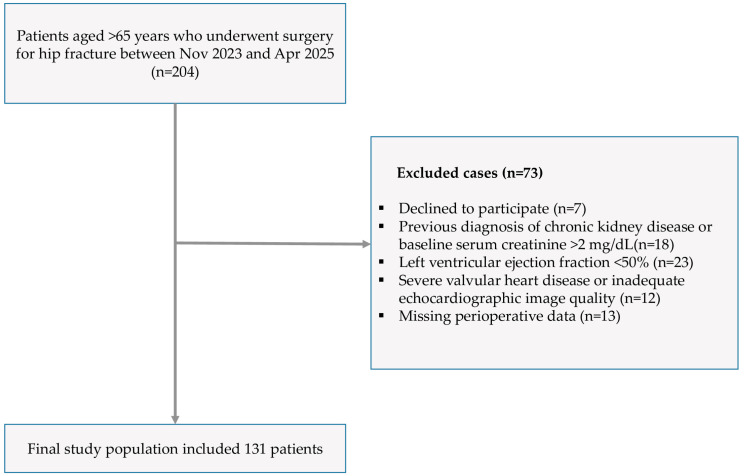
Flow diagram illustrating patient selection and exclusion criteria.

Patients under the age of 65, with a LVEF <50% on preoperative transthoracic echocardiography (TTE), severe valvular disease or inadequate echocardiographic imaging, a previous diagnosis of CKD, or a baseline creatinine level >2 mg/dL were not included in the study. Patients who declined to participate and those with missing perioperative data were also excluded. Ultimately, this study was conducted with the remaining 131 patients. Figure 1 illustrates the flow diagram for the selection and exclusion criteria for patients in this study.

Demographic data, comorbidities, baseline medications, and laboratory results were recorded. Baseline renal function parameters, including serum creatinine and eGFR, were documented. eGFR was calculated using the CKD epidemiology collaboration (CKD-EPI) formula [21].

All patients were evaluated with TTE (Vscan Extend, GE HealthCare, Milwaukee, WI, USA) in the preoperative period. To ensure measurement consistency and to reduce inter-observer variability, all TTE examinations were performed by a single experienced cardiologist using standardized acquisition protocols in accordance with current guideline recommendations [22]. During TTE, the parasternal long-axis view was primarily used. At end-diastole, the interventricular septum (IVS) and posterior wall thickness (PWT) were measured at the level of the mitral chordae. Left ventricular end-diastolic diameter (LVEDD), PWT, IVS thickness, left atrium (LA) diameter, aortic root diameter, and LVEF were recorded. RWT was calculated using the formula:RWT = 2 × PWT/LVEDD [17]. Abnormal left ventricular wall thickness was defined using established reference values, including a RWT ≥ 0.42 for both sexes and a left ventricular mass index (LVMI) > 115 g/m^2^ in men and >95 g/m^2^ in women. Left ventricular geometric patterns were classified according to the combination of RWT and LVMI as concentric hypertrophy, concentric remodeling, or normal geometry. Elevated RWT with increased LVMI was identified as concentric hypertrophy, elevated RWT with normal LVMI was identified as concentric remodeling, and a normal RWT with normal LVMI was considered normal geometry [23]. Valvular diseases (tricuspid, mitral, and aortic) were also noted, including severity.

Baseline vital signs were recorded in the premedication unit before the operation. During surgery, blood pressure was measured by an anesthesiologist at 5-min intervals using both a non-invasive cuff and an arterial line. Perioperative hypotension refers to mean arterial pressure <65 mmHg lasting for at least 10 min intraoperatively [24].

Intraoperative variables, such as type of anesthesia, case length, volume of crystalloid administration, hypotension status, estimated blood loss, and total urine output, were recorded. Patients were monitored for at least 48–72 h postoperatively. Serum creatinine levels were measured 48 h after the surgery and 7 days later if hospitalization continued. AKI was assessed according to the Kidney Disease: Improving Global Outcomes (KDIGO) criteria and was defined as an increase in serum creatinine ≥0.3 mg/dL within 48 h or ≥50% from baseline within 7 days after surgery [19,24]. AKI classification was based solely on serum creatinine criteria, as hourly urine output data were not available. Also, patients were closely monitored throughout their postoperative hospital stay for potential complications and clinical outcomes.

Written informed consent was obtained from the patients or parents and/or legal guardians of the patients. This study was conducted in accordance with the Declaration of Helsinki and was approved by The Clinical Research Ethics Committee of the Dr Abdurrahman Yurtaslan Ankara Oncology Training and Research Hospital (Approval Code: 2023-08/320, date: 23 August 2023).

## 3. Statistical Analysis

Continuous variables were described using either the mean ± standard deviation (SD) or the median and interquartile range (IQR), based on the normality of their distribution. The Shapiro–Wilk test was employed to evaluate whether the continuous variables followed a normal distribution. Categorical variables were reported as counts and percentages.

The Mann–Whitney U test was used to compare two independent groups when the variables did not follow a normal distribution. For paired samples, such as preoperative and postoperative creatinine levels, the Wilcoxon signed-rank test was used. Categorical variables were compared using the chi-square test or Fisher’s exact test, as appropriate.

Receiver operating characteristic (ROC) curve analysis was conducted to evaluate the predictive value of preoperative IVS, PWT, LA diameter, and RWT for postoperative AKI. The area under the curve (AUC), along with the 95% confidence intervals (CIs), was computed. The optimal cutoff value for RWT was determined using the Youden index derived from the ROC curve analysis and was used for exploratory subgroup analyses rather than for definitive clinical classification.

Multivariable logistic regression analysis was performed using the Enter method. Covariates were selected based on their established clinical relevance and prior evidence from the literature, given their potential effects on perioperative hemodynamics, renal perfusion, and baseline renal vulnerability to injury [19,20]. The final model included age, hypertension, diabetes mellitus, coronary artery disease, baseline medications (angiotensin-converting enzyme inhibitors/angiotensin receptor blockers, beta-blockers, loop diuretics, and oral antidiabetics), anesthesia type, and RWT. Odds ratios (ORs) with 95% CIs were reported. Additionally, RWT was analyzed as a continuous variable in a separate multivariable logistic regression model to assess the robustness of the association. To ensure clinical interpretability and model stability, the OR for continuous RWT was expressed per 0.1-unit increase. A *p*-value under 0.05 was deemed statistically significant. Statistical analyses were carried out using the Statistical Package for the Social Sciences (SPSS) version 24.0 (IBM Corp. Armonk, NY, USA).

## 4. Results

A total of 131 patients with a mean age of 80.2 ± 7.9 years were included in this study; 42% of the participants were male. A history of diabetes mellitus and hypertension was present in 34% and 59% of the patients, respectively. The mean follow-up duration was 179.4 ± 156.8 days, with a median follow-up of 122 days [IQR: 55–321]. The median LVEF was 62.0% [IQR: 60.0–64.0] and the median preoperative RWT was 0.51 [IQR: 0.45–0.57]. Based on left ventricular geometric classification, 9 patients (6.9%) had normal geometry, 58 patients (44.3%) had concentric hypertrophy, and 64 patients (48.9%) had concentric remodeling. The distribution of American Society of Anesthesiologists (ASA) physical status classifications among the patients was 16.4% ASA II, 43.1% ASA III (0.06% ASA IIIe), and 40.5% ASA IV (0.1% ASA IVe).

Laboratory parameters showed a mean baseline serum creatinine level of 0.98 ± 0.36 mg/dL and a baseline eGFR of 70.8 ± 24.6 mL/min/1.73 m^2^. Baseline eGFR showed substantial variability, and a subset of patients had values consistent with CKD stage III. Postoperative 48-h creatinine increased to 1.06 ± 0.51 mg/dL; however, this change was not statistically significant (*p* = 0.067, Wilcoxon signed-rank test). In a sensitivity analysis excluding patients with baseline eGFR <60 mL/min/1.73 m^2^, the direction of the association between increased RWT and AKI remained consistent with the primary analysis; however, statistical significance was attenuated due to the reduced sample size and limited number of AKI events. Baseline demographic and clinical characteristics and the laboratory and echocardiographic parameters of the patients are presented in Table 1.

In the postoperative period, AKI occurred in 19.1% of patients (*n* = 25), in-hospital mortality was 8.5% (*n* = 11), delirium developed in 7.6% (*n* = 10), malignant ventricular arrhythmias occurred in 3.1% (*n =* 4), new-onset atrial fibrillation (AF) was detected in 6.1% (*n =* 8), acute ischemic stroke was observed in 2.3% (*n =* 3), and acute coronary syndrome was diagnosed in 3.1% (*n =* 4). The intensive care unit (ICU) requirement was 77.1% (*n* = 101) and the prolonged length of stay (LOS) rate was 14.1% (*n =* 15).

Subgroup analyses comparing patients with RWT <0.435 and ≥0.435 showed no significant differences in AKI, mortality, delirium, ICU admission rates, cardiac complications, or ischemic events (all *p* > 0.05). No significant correlations were found between RWT and ICU mortality, in-hospital mortality, or length of hospital stay. Although certain postoperative adverse clinical outcomes appeared numerically more frequent in patients with increased RWT, these differences were not statistically significant; however, anticoagulant use was significantly more common in the RWT <0.435 group (26.3% vs. 8%, *p* = 0.032) (Supplementary Table S1).

When RWT values were compared between patients with and without adverse outcomes, no significant differences were observed with respect to all-cause mortality, in-hospital mortality, need for hemodialysis, or perioperative hypotension. However, patients who developed postoperative AKI had significantly higher RWT values compared to those without AKI (0.54 vs. 0.503, *p* = 0.048).

Echocardiographic data showed no significant differences in LVEF, IVS thickness, PWT, LA diameter, left ventricular mass (LVM), LVMI, or RWT between survivors and patients who experienced in-hospital mortality. Likewise, no differences were observed between patients who developed delirium and those who did not (all *p* > 0.05). Conversely, comparison between patients who developed AKI and those who did not revealed significantly higher values of IVS thickness (*p* = 0.024), PW thickness (*p* = 0.040), LA diameter (*p* = 0.016), and RWT (*p* = 0.048) in the AKI group. Although LVM and LVMI were higher in patients with AKI, these differences did not reach statistical significance (*p* = 0.100 and *p* = 0.061, respectively) (Table 2).

**Figure 2:**
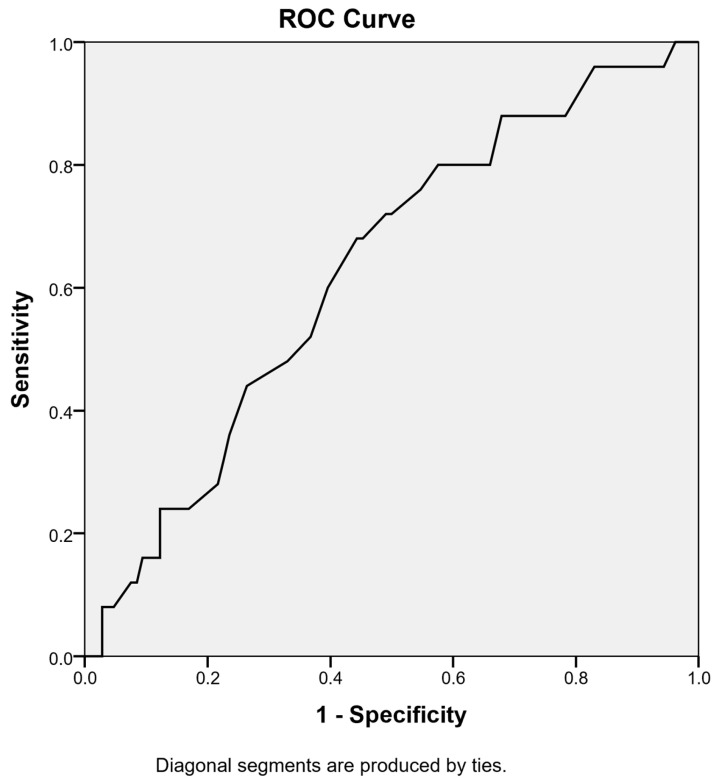
Receiver operating characteristic curve of preoperative relative wall thickness for predicting postoperative acute kidney injury. ROC, receiver operating characteristic.

The predictive value of preoperative RWT was evaluated using ROC curve analysis. RWT demonstrated a statistically discriminative predictive power for AKI development (AUC: 0.628; 95% CI: 0.512–0.743; *p* = 0.048) (Figure 2). The optimal cutoff value determined by ROC analysis was 0.435, with a sensitivity of 96.0% and a specificity of 17.0%. ROC curve analysis was also performed for IVS, PWT, and LA diameter, to evaluate their predictive ability for AKI. The AUC values were 0.645 for IVS, 0.632 for PWT, and 0.713 for LA diameter (all *p* < 0.05). The optimal cut-off values were determined using the Youden index. Although LA diameter had the highest AUC, RWT showed the highest sensitivity (96%) at the cut-off value of 0.435.

In multivariable logistic regression analysis, RWT showed a trend towards association with AKI, although it did not reach statistical significance (OR: 5.56, 95% CI: 0.74–41.45; *p* = 0.124). Other clinical variables, medication use, and anesthesia type, were not significantly associated with AKI (Table 3). In an exploratory analysis treating RWT as a continuous variable, each 0.1 unit increase in preoperative RWT was associated with a 1.5-fold increase in the odds of postoperative AKI (OR: 1.502, 95% CI: 0.765–2.948, *p* = 0.237) (Supplementary Table S2). In this continuous model, the Hosmer–Lemeshow test suggested suboptimal calibration (*p* = 0.005), likely reflecting the limited number of outcome events relative to the number of covariates; thus, these results should be interpreted as hypothesis-generating.

Comparison of preoperative and postoperative features between patients with and without AKI showed some differences. No significant differences were found in sex, hypertension, diabetes, history of stroke, coronary artery disease, Alzheimer’s disease, or AF (all *p* > 0.05). Loop diuretic use was significantly more common in patients who developed AKI (12.0% vs. 1.9%; *p* = 0.048). Regarding postoperative outcomes, in-hospital mortality was significantly higher in the AKI group (20% vs. 5.7%; *p* = 0.036). Other mortality types, delirium incidence, ICU requirement, cardiac complications, and ischemic events did not differ significantly (Supplementary Table S3).

Table 4 shows the comparison of intraoperative parameters between the groups that developed AKI and those that did not. Intraoperative crystalloid volume was significantly lower in patients who developed AKI compared to those without AKI [median (IQR): 1300 (1000–1800) mL vs. 1500 (1275–2000) mL, *p* = 0.044], whereas intraoperative urine output did not differ significantly between the groups (*p* = 0.354). Intraoperative hypotension occurred in 48 patients (36.6%). No significant difference was observed between the groups in terms of intraoperative hypotension (AKI group: *n =* 11, 44.0% vs. non-AKI group: *n =* 37, 34.9%; *p* = 0.369).

## 5. Discussion

RWT is an important marker of left ventricular structural remodeling, and increased RWT has been associated with reduced stroke volume and damage to extracardiac target organs [25,26]. This includes decreased renal function [27]. The association between this easily measured preoperative echocardiographic parameter and postoperative AKI has been previously examined in different patient populations [19,20].

Goeddel at al. retrospectively investigated the relationship between preoperative RWT and postoperative AKI in patients with preserved LVEF, undergoing noncardiac intrathoracic or intra-abdominal surgery. They found that each 0.1-unit increase in RWT was associated with approximately a 26% higher likelihood of developing AKI, and they concluded that increased RWT may be associated with subclinical cardiovascular compromise in the perioperative phase, which may lead to decreased renal perfusion and AKI [19]. Another retrospective study evaluated the predictive value of RWT for AKI among 170 patients who underwent thoracoscopic surgery for lung cancer [20]. RWT was found to be significantly associated with AKI, alongside age and mean arterial pressure. ROC analysis showed that RWT had a strong predictive ability for AKI, suggesting its usefulness in perioperative risk stratification [20]. In line with these studies in the literature, our study demonstrated that preoperative RWT was associated with postoperative AKI in elderly hip fracture patients. Although RWT demonstrated a statistically significant association with postoperative AKI, its discriminative performance was modest, with limited specificity. Therefore, RWT should not be interpreted as a standalone predictor of renal outcomes. Instead, our findings suggest that RWT may contribute to perioperative risk stratification when integrated with clinical characteristics, hemodynamic variables, and biochemical markers, particularly in elderly patients with preserved LVEF. The high sensitivity of 96.0% obtained with the specified threshold value of 0.435 suggests that RWT may be a clinically valuable parameter in excluding the risk of AKI. The cutoff value for RWT identified in this study (0.435) should be considered exploratory and requires external validation before clinical implementation. Unlike previous reports, there may be several factors that could explain why RWT had limited specificity in predicting postoperative AKI in our study. First, the exclusive inclusion of patients aged 65 years and older may have introduced age-related physiological and structural cardiac changes, potentially attenuating the prognostic value of RWT in this population [28,29]. Accordingly, the modest discriminative ability of RWT (AUC: 0.628) should be interpreted in the context of the advanced age and clinical heterogeneity of the study population. Importantly, in contrast to earlier retrospective studies, the prospective design of our study represents a methodological strength, allowing for standardized data collection and systematic preoperative assessment, which may also explain the differing results.

In several previous studies, a significant association between RWT and some clinical outcomes has been shown; for example, RWT was reported to be a reliable predictor of overall mortality and major adverse cardiac events in individuals with myocardial infarction [30]. A study showed that RWT was independently associated with the occurrence of ischemic stroke in patients with nonvalvular AF, and the role of left ventricular morphology on thromboembolic complications was emphasized [31]. Another study showed that decreased RWT values increased the risk of ventricular arrhythmia and sudden cardiac death [32]. In addition, it was stated that RWT was an effective marker for predicting cardiovascular events in hypertensive individuals with type 2 diabetes, independent of LVM [33]. In this context, our study is unique and noteworthy as it is the first prospective study in the literature to investigate the prognostic role of preoperative RWT in patients undergoing surgery for elderly hip fractures. In our study, although postoperative all-cause mortality, in-hospital mortality, ICU requirement, malignant ventricular arrhythmia, new-onset AF, and ischemic stroke rates increased in the group with RWT ≥ 0.435, they did not reach statistically significant levels. At this point, we would like to note that the high postoperative ICU admission rate observed may be related to local institutional clinical practice and postoperative management protocols. Our study revealed that the expected relationship between increased RWT and adverse clinical outcomes was not confirmed in this specific patient group. When interpreting this result, it should be taken into account that elderly patients generally exhibit more severe left ventricular hypertrophy and more pronounced diastolic dysfunction compared with younger individuals, and that age-related cardiac changes may not draw clear boundaries between “physiological” variations and “pathological” conditions [28,29]. These findings suggest that the classical prognostic value of left ventricular geometry indicators may be weakened in the elderly trauma population or that different pathophysiological mechanisms may be at the forefront [28,29]. The high sensitivity of RWT at the identified cutoff value suggests a potential role in ruling out low-risk patients rather than serving as a standalone diagnostic marker. Therefore, RWT may be more useful as part of a multimodal risk stratification strategy when combined with clinical and biochemical parameters.

Another important finding in our study was the higher use of anticoagulants in the low RWT subgroup. This likely represents differences in baseline thromboembolic risk and clinical indications (such as AF) rather than a causal link between anticoagulation and RWT. Decreased RWT is typically associated with maladaptive left ventricular remodeling, left atrial enlargement, and diastolic dysfunction. These are structural and functional changes that predispose to AF and, consequently, a greater need for anticoagulation [34].

**Figure 3:**
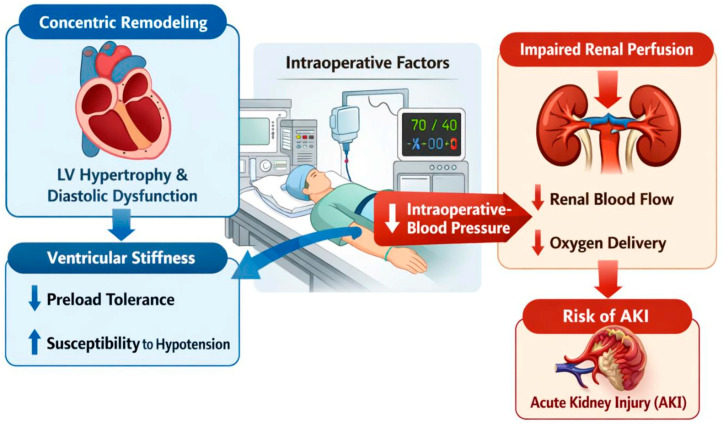
The cardio-renal mechanism between concentric remodeling and postoperative acute kidney injury. LV, left ventricular; AKI, acute kidney injury.

Within a cardio-renal framework, concentric remodeling is associated with increased ventricular stiffness and impaired diastolic filling, resulting in reduced tolerance to preload changes and limited hemodynamic reserve. During intraoperative stressors, such as hypotension and fluid shifts, this vulnerability may lead to impaired renal perfusion and increased susceptibility to postoperative AKI [35,36]. The proposed pathophysiological link between concentric remodeling and postoperative AKI is summarized in Figure 3. In the current study, we examined the relationship between the postoperative AKI and cardiac structural parameters in elderly patients undergoing surgery for hip fractures. We found that IVS thickness, PW thickness, LA diameter, and RWT were significantly higher in patients who developed AKI. Furthermore, LVMI and LVM were also higher in those who developed AKI, although these differences were not statistically significant. Importantly, our findings support the concept of a cardio-renal phenotype rather than the prognostic role of a single echocardiographic marker. The higher RWT, increased IVS thickness, PWT, and LA diameter observed in patients who developed postoperative AKI indicate a broader burden of structural cardiac remodeling. This suggests that postoperative AKI in elderly hip fracture patients may be related to impaired cardiac compliance and reduced hemodynamic reserve associated with concentric remodeling, rather than being driven by RWT alone. A similar study in the literature evaluated the relationship between certain echocardiographic parameters and postoperative AKI in elderly hip fracture patients, and reported that cardiac structural changes such as increased LVMI, enlarged LA diameter, and reduced LVEF were significantly associated with the development of AKI [37]. However, differing from that study, our study evaluated the RWT parameter for the first time and demonstrated its association with the development of postoperative AKI in this specific patient group. When the findings of both studies are evaluated together, it can be concluded that the impact of cardiac structure and left ventricular geometry on renal prognosis is non-negligible; therefore, preoperative cardiac evaluations may be an important tool in predicting the risk of AKI. In patients at higher risk for AKI based on preoperative echocardiographic findings, targeted strategies—such as optimizing volume status, careful perioperative fluid and vasopressor management, and avoiding nephrotoxic agents—may help reduce renal injury and improve postoperative outcomes [38].

Another relevant aspect that merits consideration in this context is the role of systemic hypertension. Hypertension is a major determinant of left ventricular remodeling and structural alterations, including increased RWT and myocardial stiffness. Chronic pressure overload promotes concentric myocardial adaptation and diastolic dysfunction, leading to reduced hemodynamic reserve and a limited ability to tolerate acute physiological stressors. This hemodynamic vulnerability may further predispose elderly patients with concentric remodeling to impaired renal perfusion during the perioperative period, thereby increasing susceptibility to postoperative AKI within a cardio-renal phenotype framework [39,40]. However, in the present study, the prevalence of hypertension was comparable between patients who developed AKI and those who did not, and hypertension was not identified as an independent predictor of AKI in multivariable analysis. These findings suggest that structural cardiac remodeling, rather than hypertension per se, may play a more prominent role in modulating perioperative renal vulnerability in this population.

The final point we would like to emphasize is the comparison of intraoperative data and loop diuretic use between patients who developed AKI and those who did not. Among the study groups, total amount of crystalloid administered intraoperatively was significantly lower and the frequency of loop diuretic use was higher in patients who developed AKI. Lower intraoperative fluid volume may contribute to renal hypoperfusion and ischemic tubular injury, consistent with previous evidence showing that restrictive fluid strategies increase the risk of postoperative AKI, particularly in the elderly and those who are hemodynamically fragile [41]. The higher rate of diuretic use among patients who developed AKI may indicate preexisting fluid imbalance, diuretic dependence, or attempts to manage perioperative congestion, all of which have been linked to greater renal vulnerability [42]. These findings highlight the importance of accurately identifying patients at risk for AKI in the preoperative period, meticulously optimizing fluid management during surgery, and reconsidering the necessity of diuretic use, especially in elderly patients.

## 6. Limitations

This study has several limitations. First, the relatively small number of AKI events (*n* = 25) may have reduced statistical power and widened confidence intervals. In addition, the limited number of AKI events precluded the reliable inclusion of interaction terms between RWT, fluid administration, and hypotension in multivariable models. Although intraoperative urine output was recorded, AKI was defined solely based on serum creatinine criteria. The lack of hourly postoperative urine output monitoring prevented the full application of KDIGO urine-output staging, which may limit the sensitivity of AKI detection. Another limitation is the low specificity of RWT in predicting postoperative AKI. The low specificity of RWT limits its use as a standalone predictor and supports its interpretation within a multimodal risk stratification framework. Detailed data on perioperative nonsteroidal anti-inflammatory drug (NSAID) exposure and contrast media use were not systematically collected, and patients exposed to high-risk drug combinations (such as angiotensin-converting enzyme inhibitor/angiotensin receptor blocker, NSAID, and diuretic use) were not excluded, which may have introduced residual confounding. Although the use of well-established echocardiographic parameters and standardized measurement techniques supports the reproducibility of our findings, intra-observer variability analyses were not formally assessed, which may limit the evaluation of measurement reproducibility. The heterogeneous baseline renal function of the study population, including patients with reduced eGFR, may have influenced AKI development independently of echocardiographic parameters. Despite excluding patients with advanced renal dysfunction, individuals with mild CKD (eGFR < 60 mL/min/1.73 m^2^) may have been included, potentially introducing residual confounding. The sensitivity analysis excluding patients with baseline eGFR < 60 mL/min/1.73 m^2^ was limited by a reduced number of AKI events, leading to model instability and wide confidence intervals. Consequently, effect estimates for RWT could not be reliably quantified in this subgroup, and these results should be interpreted cautiously. Detailed time-weighted analyses of intraoperative hypotension and continuous fluid balance could not be performed due to the lack of granular hemodynamic data, which may have limited the assessment of perioperative contributors to AKI. Comprehensive assessment of diastolic function, including left atrial volume index, was not possible due to unavailable measurements, which may have limited the evaluation of additional echocardiographic predictors of AKI. Furthermore, although RWT was analyzed as a continuous variable, the limited number of AKI events relative to the number of covariates restricted our ability to perform complex non-linear modeling, such as restricted cubic splines or internal validation through bootstrapping, due to the risk of model overfitting. Finally, the single-center design and high ICU admission rate may reflect local practice patterns and limit the generalizability of our findings.

Despite these limitations, our study also has strengths. We believe our study makes a modest but significant contribution by presenting data from a specific and underrepresented patient population, such as elderly patients undergoing hip fracture surgery. We hope that these findings will serve as a foundation for future studies with larger scales, more robust methodological designs, and more detailed perioperative renal monitoring in this vulnerable and clinically important cohort.

## 7. Conclusions

Preoperative RWT demonstrated a modest but statistically significant association with postoperative AKI in elderly hip fracture patients with preserved LVEF. The prognostic value of left ventricular geometric parameters may be attenuated in the elderly, possibly due to differing underlying pathophysiological mechanisms. RWT may represent a complementary echocardiographic marker within a broader multimodal framework for perioperative risk stratification rather than a standalone predictor of postoperative AKI.

## Figures and Tables

**Table 1:** Baseline demographic, clinical, laboratory, echocardiographic, and perioperative characteristics of the study population.

Descriptive Statistics			
*n* = 131	Median	IQR	Mean ± Std. Deviation
Age, year	80	13	80.2 ± 7.8
Height, cm	160	15	162.8 ± 9.3
Weight, kg	74	17	73.8 ± 13.7
BMI, kg/m^2^	24.34	6.41	27.8 ± 4.8
BSA_H, m^2^	1.65	0.46	1.67 ± 0.36
Systolic blood pressure, mmHg	120	20	122.0 ± 17.5
Diastolic blood pressure, mmHg	70	13	73.1 ± 10.3
Heart rate, min	80	16	83.1 ± 13.2
eGFR, mL/min/1.73 m^2^	71.9	37.1	70.8 ± 24.6
BUN, mg/dL	21.5	11.9	29.1 ± 35.1
Creatinine, mg/dL	0.89	0.53	0.98 ± 0.36
Sodium, mmol/L	137	3.0	137.2 ± 3.0
Potassium, mmol/L	4.2	0.7	4.1 ± 0.5
Hemoglobin, g/dL	11.3	2.9	11.5 ± 1.9
48th H eGFR, mL/min/1.73 m^2^	66.1	43.7	66.1 ± 29.3
48th H serum creatinine, mg/dL	0.89	0.62	1.06 ± 0.51
LVEF, %	62	4	61.6 ± 3.3
IVS, mm	11.8	2.2	11.9 ± 1.7
PWT, mm	11.4	2.3	11.3 ± 1.5
LVEDD, mm	43.3	4.6	43.8 ± 3.6
LA diameter, mm	36	9	36 ± 5.8
LV mass, g	176	50	181 ± 44.8
LVMI (g/m^2^)	96	24	100 ± 21.5
RWT	0.51	0.12	0.521 ± 0.085
Case length, min	135	50	143 ± 63.8
Total crystalloid administered, mL	1500	800	1615 ± 953
Total blood transfusion, mL	0.00	0	63.9 ± 183.1
Urine output, mL	100	100	150 ± 143.9
Total ICU LOS, days	1.0	0	2.3 ± 6.9
Total hospital LOS, days	2.0	2	4.1 ± 5.1

Values are presented as mean ± standard deviation unless otherwise specified. BMI, body mass index; BSA_H, body surface area estimated based on height; eGFR, estimated glomerular filtration rate; BUN, blood urea nitrogen; LVEF, left ventricular ejection fraction; IVS, interventricular septum; PWT, posterior wall thickness; LVEDD, left ventricular end-diastolic diameter; LV, left ventricular; LA, left atrium; LVMI, left ventricular mass index; RWT, relative wall thickness; ICU LOS, intensive care unit length of stay; LOS, length of stay.

**Table 2:** Comparison of echocardiographic parameters between patients with and without AKI.

Variable	No. AKI (*n* = 106) Median [IQR]	AKI (*n* = 25) Median [IQR]	Mann–Whitney U	*p*-Value
LVEF, %	62.0 [60.0–65.0]	61.0 [60.0–63.0]	1243.0	0.621
IVS, mm	11.6 [10.7–12.6]	12.5 [11.4–13.4]	941.0	**0.024**
PWT, mm	11.3 [10.1–12.2]	12.3 [10.4–12.8]	975.0	**0.040**
LA diameter, mm	35.1 [31.0–39.3]	38.7 [35.4–43.9]	250.0	**0.016**
LVEDD, mm	43.3 [41.3–48.8]	43.7 [41.9–45.4]	1295.0	0.860
LV mass, g	175.8 [150.4–198.3]	182.5 [161.1–234.8]	1044.5	0.100
LVMI, g/m^2^	94.5 [85.5–108.8]	104.7 [89.3–122.9]	1005.0	0.061
RWT	0.503 [0.450–0.560]	0.54 [0.495–0.605]	987.0	**0.048**

AKI, acute kidney injury; LVEF, left ventricular ejection fraction; IVS, interventricular septum; PWT, posterior wall thickness; LVEDD, left ventricular end-diastolic diameter; LA, left atrium; LV, left ventricle; LVMI, left ventricular mass index; RWT, relative wall thickness; IQR, interquartile range. Values are presented as median [25th–75th percentile]. *p*-values derived from Mann–Whitney U test. Statistically significant values are indicated in bold.

**Table 3:** Multivariable logistic regression analysis for predictors of AKI.

Variable	OR	95% CI	*p*-Value
Age	1.01	0.94–1.07	0.766
Hypertension	0.31	0.06–1.64	0.150
Diabetes Mellitus	0.22	0.02–2.56	0.233
Coronary Artery Disease	0.57	0.12–2.61	0.521
ACE/ARB	2.34	0.51–10.68	0.264
Beta Blocker	2.60	0.77–8.79	0.143
Loop Diuretic	3.66	0.40–33.49	0.289
Oral Antidiabetics	5.30	0.43–64.66	0.211
RWT ≥ 0.435	5.56	0.74–41.45	0.124
General Anesthesia	1.73	0.49–6.07	0.377

AKI, acute kidney injury; OR, Odds ratio, CI, confidence interval; ACE/ARB, angiotensin-converting enzyme inhibitor/angiotensin receptor blocker; RWT, relative wall thickness. Note: Model calibration was assessed using the Hosmer–Lemeshow goodness-of-fit test (χ^2^ = 9.94, *p* = 0.269). Model discrimination was evaluated using Nagelkerke R^2^ (0.160).

**Table 4:** Intraoperative parameters according to the development of AKI.

Variable	No. AKI (*n* = 106) Median [IQR]	AKI (*n* = 25) Median [IQR]	Mann–Whitney U	*p*-Value
Case length, min	135 [108–161]	120 [108–155]	1171.5	0.878
Total crystalloid administration	1500 [1275–2000]	1300 [1000–1800]	985.0	**0.044**
Blood loss, mL	300 [300–500]	300 [275–500]	1298.0	0.872
Blood transfusion, mL	0 [0–0]	0 [0–0]	1220.0	0.694
Urine output, mL	100 [100–200]	100 [67–200]	1159.0	0.354

AKI: acute kidney injury. Values are presented as median [interquartile range, IQR: 25th–75th percentile]. *p*-values were calculated using the Mann–Whitney U test. Most patients did not receive an intraoperative blood transfusion; therefore, the median value is 0, with no interquartile range. This reflects a highly skewed distribution, and the median [IQR] is preferred over the mean in such cases. Statistically significant values are indicated in bold.

## Data Availability

The data presented in this study are available on request from the corresponding author.

## Supplementary Materials

The following supporting information can be downloaded at: https://www.mdpi.com/article/10.3390/jcm15031115/s1, Table S1: Comparison of RWT units with demographic and clinical outcomes of patients (cut-off point: 0.435), Table S2: Multivariable logistic regression analysis with RWT modeled as a continuous variable, Table S3: Comparison of preoperative patient characteristics and postoperative outcomes according to the presence of AKI.
