# Supplementary material for: Evaluation of Preoperative Left Ventricular Relative Wall Thickness for Predicting Postoperative Acute Kidney Injury in Elderly Hip Fracture Patients
Source: J Clin Med. 2026 Jan 30;15(3):1115. doi: 10.3390/jcm15031115 (PMC12898542; doi:10.3390/jcm15031115)
Supplement: Supplementary file 1 [file jcm-15-01115-s001.zip › jcm-4093192-supplementary.pdf]

**Supplementary Table S1. Comparison of RWT units with demographic and clinical outcomes of patients (cut-off point: 0.435)**

| | All patients (n=131) | RWT < 0.435 n=19, (%) | RWT ≥ 0.435 n=112, (%) | p value |
|---|---|---|---|---|
| Gender, male | 55 (42.0) | 8 (42.1) | 47 (42.0) | 1.000 |
| Hypertension | 78 (59.5) | 14 (73.7) | 64 (57.1) | 0.269 |
| Diabetes mellitus | 44 (33.6) | 7 (36.8) | 37 (33.0) | 0.950 |
| Ischemic stroke | 12 (9.2) | 1 (5.3) | 11 (10.7) | 0.689 |
| Coronary artery disease | 25 (19,7) | 6 (31.6) | 19 (17.6) | 0.208 |
| Alzheimer | 17 (13.8) | 2 (11.1) | 15 (14.3) | 1.000 |
| Atrial fibrillation | 8 (6.8) | 2 (10.5) | 6 (6.1) | 0.613 |
| 48$^{th}$ hour eGFR<60 mL/min/1, 73 m$^2$ | 58 (44.2) | 7 (36.8) | 51 (45.5) | 0.649 |
| ACEI/ARB use, n (%) | 56 (45.9) | 12 (66.7) | 44 (42.3) | 0.097 |
| Beta blocker use | 41 (31.3) | 9 (47.4) | 32 (28.6) | 0.172 |
| Loop diuretic use | 5 (3.8) | 0 (0.0) | 5 (4.5) | 1.000 |
| Thiazide diuretic use | 26 (19.8) | 5 (26.3) | 21 (18.8) | 0.533 |
| Calcium channel blocker use | 46 (35.1) | 7 (36.8) | 39 (34.8) | 1.000 |
| Statin use | 17 (13.0) | 3 (15.8) | 14 (12.5) | 0.713 |
| Anticoagulant use | 14 (10.7) | 5 (26.3) | 9 (8.0) | **0.032** |
| Antiplatelet use | 37 (28.3) | 6 (31.6) | 31 (27.7) | 0.787 |
| OAD use | 29 (22.1) | 5 (26.3) | 24 (21.4) | 0.765 |
| Alpha blocker use | 3 (2.3) | 1 (5.3) | 2 (1.8) | 0.378 |
| Mortality, all | 41 (31.3) | 4 (21.4) | 37 (33.0) | 0.439 |
| Mortality, hospital | 11 (8.5) | 1 (5.3) | 10 (9.0) | 1.000 |
| Delirium | 10 (7.6) | 2 (10.5) | 8 (7.1) | 0.638 |
| Acute kidney injury | 25 (19.1) | 1 (5.3) | 24 (21.4) | 0.122 |
| ICU requirement | 101 (77.1) | 14 (73.7) | 87 (77.7) | 0.769 |
| Haemodialysis | 3 (2.3) | 1 (5.3) | 2 (1.8) | 0.378 |
| Malignant ventricular arrhythmia | 4 (3.1) | 0 (0.0) | 4 (3.6) | 1.000 |
| De Novo atrial fibrillation | 8 (6.1) | 1 (5.3) | 7 (6.3) | 1.000 |
| Post operative ischemic stroke | 3 (2.3) | 0 (0.0) | 3 (2.7) | 1.000 |
| MINS | 12 (9.2) | 2 (10.5) | 10 (8.9) | 0.686 |
| Acute coronary syndrome | 4 (3.1) | 1 (5.3) | 3 (2.7) | 0.470 |

Values are presented as number (%). RWT, relative wall thickness; eGFR,estimated glomerular filtration rate; ACEI,angiotensin-converting enzyme inhibitor; ARB,angiotensin receptor blocker; OAD,oral antidiabetic; ICU, intensive care unit; MINS,myocardial injury after non-cardiac surgery.Statistically significant values are indicated in bold.

**Supplementary Table S2 . Multivariable logistic regression analysis with RWT modeled as a continuous variable.**

| **Variable** | **OR** | **95% CI** | **p-value** |
|---|---|---|---|
| RWT (per 0.1 unit increase) | 1.502 | 0.765 – 2.948 | 0.237 |
| Age | 1.040 | 0.974 – 1.110 | 0.237 |
| Hypertension | 0.331 | 0.071 – 1.544 | 0.159 |
| Diabetes mellitus | 0.308 | 0.029 – 3.320 | 0.332 |
| Coronary artery disease | 0.453 | 0.084 – 2.453 | 0.358 |
| ACE/ARB use | 2.118 | 0.502 – 8.931 | 0.307 |
| Beta-blocker use | 2.231 | 0.644 – 7.734 | 0.206 |
| Loop diuretics | 7.449 | 0.605 – 91.649 | 0.117 |
| Oral antidiabetics | 4.543 | 0.346 – 59.691 | 0.249 |
| General anesthesia | 1.666 | 0.513 – 5.411 | 0.396 |

RWT, relative wall thickness ; ACEI,angiotensin-converting enzyme inhibitor; ARB,angiotensin receptor blocker .In the exploratory continuous-RWT model, calibration assessed by the Hosmer–Lemeshow test suggested suboptimal fit ($p=0.005$), likely reflecting the limited number of outcome events relative to the number of covariates. Therefore, this analysis should be interpreted as hypothesis-generating.

**Supplementary Table S3 . Comparison of preoperative patient characteristics and postoperative outcomes according to the presence of AKI**

| | All patients (n=131) | AKI (+), n=25 | AKI (-), n=106 | p value |
|---|---|---|---|---|
| Gender, male | 55 (42.0) | 11 (44.0) | 44 (41.5) | 0.999 |
| Hypertension | 78 (59.5) | 13 (52.0) | 65 (61.3) | 0.530 |
| Diabetes mellitus | 44 (33.6) | 6 (24.0) | 38 (35.3) | 0.372 |
| Ischemic stroke | 12 (9.2) | 2 (8.3) | 10 (10.2) | 1.000 |
| Coronary artery disease | 25 (19,7) | 3 (12.0) | 22 (21.6) | 0.402 |
| Alzheimer | 17 (13.8) | 3 (12.0) | 14 (13.2) | 1.000 |
| Atrial fibrillation | 8 (6.8) | 2 (8.7) | 6 (6.3) | 0.653 |
| $48^{th}$ hour eGFR<60 | 58 (44.3) | 23 (92.0) | 35 (33.0) | **< 0.001** |
| ACEI/ARB use, n (%) | 56 (45.9) | 12 (48.0) | 44 (41.5) | 0.661 |
| Beta blocker use | 41 (31.3) | 10 (40.0) | 31 (29.2) | 0.422 |
| Loop diuretic use | 5 (3.8) | 3 (12.0) | 2 (1.9) | **0.048** |
| Thiazide diuretic use | 26 (19.8) | 6 (24.0) | 20 (18.9) | 0.582 |
| Calcium channel blocker use | 46 (35.1) | 6 (24.0) | 40 (37.7) | 0.289 |
| Statin use | 17 (13.0) | 2 (8.0) | 15 (14.2) | 0.525 |
| Anticoagulant use | 14 (10.7) | 2 (8.0) | 12 (11.3) | 1.000 |
| Antiplatelet use | 37 (28.3) | 7 (28.0) | 30 (28.3) | 0.808 |
| OAD use | 29 (22.1) | 5 (20.0) | 24 (22.6) | 0.985 |
| Alpha blocker use | 3 (2.3) | 0 (0.0) | 3 (2.8) | 1.000 |
| General anesthesia | 29 (22.1) | 7 (28.0) | 22 (20.8) | 0.514 |
| Spinal anesthesia | 92 (70.2) | 15 (60.0) | 77 (72.6) | 0.446 |
| Sevorane usage | 26 (21.8) | 6 (24.0) | 20 (18.9) | 0.397 |
| Mortality, all | 41 (31.3) | 11 (44.0) | 30 (28.3) | 0.200 |
| Mortality, hospital | 11 (8.5) | 5 (20.0) | 6 (5.7) | **0.036** |
| Delirium | 10 (7.6) | 4 (16.0) | 6 (5.7) | 0.097 |
| ICU requirement | 101 (77.1) | 19 (76.0) | 82 (77.4) | 1.000 |
| Malignant ventricular arrhythmia | 4 (3.1) | 2 (8.0) | 2 (1.9) | 0.164 |
| De Novo atrial fibrillation | 8 (6.1) | 2 (8.0) | 6 (5.7) | 0.648 |
| Post operative ischemic stroke | 3 (2.3) | 1 (4.0) | 2 (1.9) | 0.473 |
| MINS | 12 (9.2) | 4 (16.0) | 8 (7.5) | 0.242 |
| Acute coronary syndrome | 4 (3.1) | 1 (4.0) | 3 (2.8) | 0.576 |

Values are presented as number (%).AKI , acute kidney injury; eGFR, estimated glomerular filtration rate; ACEI,angiotensin-converting enzyme inhibitor; ARB,angiotensin receptor blocker; OAD,oral antidiabetic; ICU,intensive care unit; MINS,myocardial injury after non-cardiac surgery.Statistically significant values are indicated in bold.
